# Association Between the Frailty Index Based on Laboratory Tests and All-Cause Mortality in Hospitalized Older Adults: Retrospective Cohort Study

**DOI:** 10.2196/70204

**Published:** 2025-09-10

**Authors:** Eyal Pasternak, Tamar Freud, Yan Press

**Affiliations:** 1 Division of Community Health, Faculty of Health Sciences, Ben-Gurion University of the Negev, Beer- Sheva, Israel; 2 Soroka Medical Center, Beer-Sheva, Israel; 3 Siaal Research Center for Family Medicine and Primary Care, Faculty of Health Sciences, Ben-Gurion University of the Negev, Beer- Sheva, Israel; 4 Department of Geriatrics, Soroka Medical Center, Ben-Gurion University of the Negev, Reger Boulevard, POB 151, Beer-Sheva, 8410101, Israel, 1 972-50-6263903; 5 Unit for Community Geriatrics, Division of Health in the Community, Ben-Gurion University of the Negev, Beer-Sheva, Israel; 6 Center for Multidisciplinary Research in Aging, Ben-Gurion University of the Negev, Beer-Sheva, Israel

**Keywords:** FI-Lab, all-cause mortality, hospitalized, older adults, risk factor

## Abstract

**Background:**

Frailty is a common issue among hospitalized older adult patients and is associated with numerous adverse health outcomes. Assessing frailty facilitates better decision-making for treatment plans, patient placement, and discharge planning. Approximately a decade ago, the frailty index based on laboratory tests (FI-Lab) metric was introduced. Although this index has been shown in numerous studies to predict adverse medical outcomes, including mortality, it has not been extensively evaluated among patients hospitalized in internal medicine departments for diverse indications.

**Objective:**

The aim of the study was to investigate the relationship between FI-Lab at admission and all-cause mortality during hospitalization and after discharge in patients aged 65 years and older admitted for diverse clinical indications to internal medicine departments.

**Methods:**

This retrospective cohort study included patients aged 65 years and older hospitalized in the internal medicine departments of a large tertiary hospital. Data included demographic variables, comorbidity, and all-cause mortality. The FI-Lab was calculated based on 16 available blood tests, as well as blood pressure and heart rate measurements. We used Cox proportional hazards regression models to evaluate associations with mortality. Model performance was assessed using the C-index and time-dependent receiver operating characteristic (ROC) curves. Hospitalization data were collected from December 25, 2016, to January 7, 2023.

**Results:**

During the study period, 31,443 patients were hospitalized in internal medicine departments, and FI-Lab was calculable for 31,398 of them. The mean age of the patients was 77.6 (SD 8.2) years, and 52.1% (16,346/31,443) were women. The mean FI-Lab score was 0.38 (SD 0.15). Based on FI-Lab scores, patients were categorized into 4 groups: robust, mildly prefrail, moderately prefrail, and frail. After adjusting for age, sex, and comorbidities, frail and prefrail patients exhibited higher mortality rates than robust patients. For each 0.01 increase in the FI-Lab score (as a continuous variable), adjusted analyses revealed a 5.5% increase in in-hospital mortality, a 2.9% increase in mortality within the first year after hospitalization, and a 1.9% increase in mortality beyond the first year.

**Conclusions:**

The FI-Lab is a readily available and informative metric of frailty in older hospitalized patients. Calculating this index can assist physicians with identifying patients at high risk of mortality and provide meaningful information to support clinical decision-making.

## Introduction

Frailty is a condition of increased vulnerability to impaired recovery of homeostasis following stress [1]. Frailty is highly prevalent among adults. In a nationally representative sample of Medicare enrollees aged 65 years and older, the prevalence of prefrail individuals was 45.5%, while the prevalence of frail individuals was 15.3% [2]. The rate of frailty among hospitalized patients is even higher. In a study of more than 22,000 individuals aged 65 years and older admitted to internal medicine departments, 43% were classified as prefrail, and 20% were classified as frail [3]. A higher prevalence was found in a meta-analysis by Ligthart-Melis et al [4], with 47% classified as prefrail and 36% classified as frail.

Among older hospitalized patients, frailty is associated with longer hospital stays [3,5-7], a higher likelihood of admission to an intensive care unit (ICU) and extended time in it [3], falls during hospitalization [6], delirium [6], pressure ulcers [6], an increased likelihood of institutionalization after discharge [3,6], a greater risk of readmission [3,8], higher hospitalization costs [9], and even increased mortality [3,5,6,10,11].

In 2001, two primary models of frailty were introduced: the cumulative deficit model [12] and the phenotype model [13]. Based on these two models, more than 65 tests have been developed to assess frailty [14]. Identifying frailty among hospitalized patients is essential as it enables, on one hand, risk stratification [7,15] and, on the other hand, the development of an intervention plan tailored for frail patients. Such interventions have been shown to shorten hospital stay [16], reduce the need for medical services after discharge, lower the likelihood of readmission, increase patient satisfaction, and even decrease mortality [17]. Despite this, frailty assessment among hospitalized patients is limited because it requires completion of a long checklist, a time-consuming process that clinicians in busy departments often cannot handle [18]. Some frailty assessment tools rely on self-reported data (eg, weight loss in the absence of objective data, information on physical activity, or feelings of exhaustion) or require patient cooperation during assessment (eg, walking speed and handgrip strength measurements) [19]. It is quite likely that such assessments would be difficult to conduct during acute hospitalization. To address these challenges, a frailty index based on laboratory tests (FI-Lab) was introduced for the first time in 2014 [20]. Since then, numerous studies have been conducted using various versions of FI-Lab. These have demonstrated its association with adverse health outcomes, including longer hospital stays [21-25]; an increased likelihood of ICU admission or prolonged ICU stays [22,23]; a greater risk for readmission [21,22,26,27]; institutionalization after discharge [24,27]; greater postdischarge care needs [21]; and increased in-hospital mortality [24,25,27-32], mortality within the first year after discharge [18,27,28,33-37], and even beyond 1 year after discharge [19,21,32,36,38-41].

As demonstrated in our literature review (see Multimedia Appendix 1), the majority of studies have focused on populations with specific medical conditions (eg, heart failure, pneumonia, sepsis). Only a limited number of studies (eg, [38,40]) have examined populations admitted to internal medicine departments for diverse indications. Moreover, to our knowledge, no studies to date have explored the relationship between FI-Lab and all-cause mortality among hospitalized patients in Israel.

The aim of this study was to evaluate the association between FI-Lab at the time of admission and mortality, both during hospitalization and following discharge, among patients aged 65 years and older admitted for diverse indications to internal medicine departments in a large tertiary care hospital in Israel.

## Methods

### Study Setting and Population

This was a retrospective cohort study using data from electronic medical records (EMRs) for patients aged 65 years and older who were hospitalized for any reason in the Internal Medicine Division at Soroka University Medical Center (SUMC) in southern Israel. SUMC is the third-largest tertiary medical center in Israel and the largest in southern Israel, with a capacity of 1200 beds. The Division of Internal Medicine consists of 7 internal medicine wards.

The study population included men and women 65 years of age and older who were hospitalized in one of the study wards for any reason during the 6-year period from December 25, 2016, to January 1, 2023. The follow-up period for mortality ended on November 16, 2024. It should be noted that, if a patient had multiple hospitalizations during the follow-up period, the first hospitalization was considered the index event.

### Data Collection

The following data were collected from the EMR: sociodemographic, clinical, laboratory, and mortality.

Sociodemographic data included age, sex, and ethnicity.

Clinical data included the primary reason for hospital admission (according to the emergency department diagnosis) and comorbid diseases. The Charlson Comorbidity Index (CCI) [42] was automatically calculated. The first measurement of systolic and diastolic blood pressures and heart rate at triage in the emergency department was recorded.

Laboratory data included venous blood tests performed during evaluations in the emergency department or within the first 48 hours of hospitalization. We focused on 16 blood tests, at least 14 of which are routinely performed in the emergency department: hemoglobin, hematocrit, mean corpuscular volume, mean corpuscular hemoglobin, white blood cells, lymphocyte number, platelets, mean platelet volume, glucose, creatinine, urea, sodium, potassium, chloride, protein, and albumin. The latter two were usually performed during hospitalization.

*All-cause mortality* was defined as in-hospital mortality, mortality within 1 year after discharge, and mortality during the entire study period. Data on all-cause mortality were obtained from the SUMC computerized system, which is regularly updated with information from the Ministry of Interior’s database.

### F1-Lab Construction

A total of 19 items were used to construct the FI-Lab, including 16 blood tests from venous blood samples and 3 vital signs recorded during triage in the emergency department: systolic blood pressure, diastolic blood pressure, and heart rate. Each item was dichotomized based on the normal reference intervals defined by the SUMC laboratory norms. Values outside the reference range were assigned a score of 1, while values within the reference range were assigned a score of 0. The FI-Lab score was calculated by summing the number of deficits present and dividing the total by the number of items included. The FI-Lab score ranged from 0 to 1, with higher scores indicating greater frailty. Only patients who had more than 85% of the necessary items (n>16) were included. The FI-Lab scores were categorized by quartiles and as a continuous variable (0.01-point increase).

### Power Analysis

To estimate statistical power, we assumed exponentially distributed survival times and relied on outcome rates from a previously published cohort study with a military veteran population [27] in which 27.5% of the high-risk group and 8.2% of the low-risk group died within 1 year. Based on these mortality rates and an expected research population of 30,000 patients, with approximately 12% classified as high risk, we estimated the hazard ratio (HR) to be 3.76.

Power analysis for time-to-event outcomes was conducted using the method for proportional hazards regression by Schoenfeld [43].

This calculation was implemented in R, using standard functions to compute the normal quantiles and logarithmic transformation of the HR. The resulting estimate showed that only 116 events were required to achieve 99% power. Given the expected >2000 events in our study, we concluded that the study was well-powered to detect a significant difference in survival outcomes.

### Statistical Analysis

Statistical analyses were performed using the SPSS version 29 (IBM Corp) and R software (version 4.4.3). A *P* value <.05 was considered statistically significant. All tests were 2-sided. Categorical variables are described as frequencies and percentiles. Continuous variables, such as age, are described as mean (SD). The comparison of specific characteristics between frailty status groups, as assessed using FI-Lab scores, was carried out using the *χ*^2^ test for categorical variables and Kruskal-Wallis test for continuous variables, such as length of hospital stay, which did not meet the assumption of normal distribution.

Cox proportional hazards assumptions were evaluated using the Schoenfeld residuals test (cox.zph function in R) and by visual inspection of log(-log[survival]) plots. No significant violations of the proportional hazards assumption were detected for the variables included in the models (global test *P*>.05).

Cox regression models were built for all-cause mortality according to frailty status, as assessed using FI-Lab scores, at 4 different points in time: in-hospital mortality, mortality during the first year after discharge, mortality beyond the first year after discharge, and mortality at any time. These models are presented as adjusted survival curves for all-cause mortality at different levels of frailty status, as assessed using FI-Lab scores, and as adjusted HRs for FI-Lab scores, age, gender, CCI scores, and the 4 main indications for hospitalization (cardiovascular, musculoskeletal, respiratory, and fever).

To evaluate the predictive performance of FI-Lab, we used the Harrell C-index (concordance index) and time-dependent receiver operating characteristic (ROC) curves based on Cox proportional hazards models. Adjusted survival curves were generated using the *adjustedCurves* package (version 0.6.2) in R (version 4.4.3) [44,45]. The analysis was based on Cox proportional hazards models fitted with the coxph() function from the *survival* package [46], and adjusted estimates were visualized using *ggplot2* [47] and the *survminer* package (version 0.4.9) [48].

Each plot illustrates the ROC curves for the multivariable model with and without the FI-Lab variable. The comparison between models with and without the FI-Lab variable was performed using time-dependent areas under the curve (AUCs), and statistical differences were tested using a bootstrap method with 2000 resamples. The AUC values are provided for both models, along with the *P* values from the statistical comparison of AUCs.

### Ethical Considerations

The study was approved by the Helsinki Committee of the Soroka Medical Center (SOR-0297‐23). The study was based on retrospective analysis of anonymized EMR data. Informed consent was not required according to national regulations and institutional review board policy. All data were handled in compliance with institutional data privacy and confidentiality standards.

## Results

### Study Population Characteristics

During the study period, 31,443 patients aged 65 years and older were hospitalized in the Internal Medicine Division at SUMC. Of these, 31,398 had sufficient data to calculate FI-Lab.

The key characteristics of the 31,443 patients for whom FI-Lab was calculated are presented in Table 1. The average age was 77.6 (SD 8.2) years, 16,346 (52.1%) were women, and 27,824 (88.7%) were Jewish. The 3 primary reasons for hospitalization, as recorded in the emergency department, were cardiovascular problems (6491/31,443, 20.7%), musculoskeletal issues (5460/31,443, 17.4%), and respiratory problems (3141/31,443, 10%). The average CCI score was 3.7 (SD 2.8).

**Table 1.** Comparison of the study population (N=31,443): survivors versus nonsurvivors.

	Survivors (n=16,260)		Nonsurvivors (n=15,138)		*P* value
Age (years), mean (SD)	74.8 (7.1)		80.7 (8.4)		<.001
Age (years), median (range)	74 (65-111)		81 (65-111)		<.001
Gender, n (%)					.003
Male	7662 (47.1)		7390 (48.8)		
Female	8598 (52.9)		7746 (51.2)		
Missing	0 (0)		2 (0.01)		
Ethnicity, n (%)					<.001
Jewish	14,261 (87.9)		13,563 (89.6)		
Arab	1963 (12.1)		1574 (10.4)		
Missing	36 (0.2)		1 (0.01)		
Primary reason for hospital admission, n (%)					<.001
Cardiovascular	3647 (22.4)		2844 (18.8)		
Musculoskeletal	3771 (23.2)		1689 (11.2)		
Respiratory	1341 (8.2)		1800 (11.9)		
Fever	1358 (8.4)		1777 (11.7)		
Infection	1057 (6.5)		1212 (8)		
Gastrointestinal	859 (5.3)		887 (5.9)		
Other	4227 (26)		4929 (32.6)		
Total CCI^a^ score, mean (SD)	2.9 (2.3)		4.5 (2.9)		<.001
Total CCI score, median (range)	2 (0‐18)		4 (0‐22)		<.001
FI-Lab score, mean (SD)	0.33 (0.14)		0.43 (0.15)		<.001
FI-Lab score, median (range)	0.31 (0‐0.89)		0.42 (0‐0.94)		<.001
Frailty status assessed using FI-Lab^b^ scores, n (%)					<.001
Robust (<0.2632)	6083 (37.4)		2545 (16.8)		
Mildly prefrail (0.2633‐0.0.3684)	3772 (23.2)		2563 (16.9)		
Moderately prefrail (0.3685‐0.4737)	4240 (26.1)		5090 (33.6)		
Frail (>0.4738)	2165 (13.3)		4940 (32.6)		
Hospitalization duration (days), mean (SD)	2.9 (6.4)		4.3 (7.6)		<.001
Hospitalization duration (days), median (range)	1 (0‐246)		2 (0‐139)		<.001
Timing of mortality, n (%)					—^c^
Died while hospitalized	—		1658 (11)		
Died in the first year	—		5174 (34.2)		
Died after 1 year	—		8306 (54.9)		

a CCI: Charlson Comorbidity Index.

b FI-Lab: frailty index based on laboratory tests.

c Not applicable.

### F1-Lab Scores

The mean FI-Lab score was 0.38 (SD 0.15), with a median of 0.37 (range 0.0-0.94).

Since the FI-Lab scores were not normally distributed, each quartile contained an unequal number of patients. The first quartile consisted of 8628 patients (8628/31,443, 27.5%) who had an FI-Lab score <0.2632 and were classified as “robust.” The second quartile consisted of 6335 patients (6335/31,443, 20.2%) who had an FI-Lab score between 0.2633 and 0.3684 and were classified as “mildly prefrail.” The third quartile consisted of 9930 patients (9930/31,443, 29.7%) who had an FI-Lab score between 0.3685 and 0.4737 and were classified as “moderately prefrail.” The fourth quartile consisted of 7105 patients (7105/31,443, 22.6%) who had an FI-Lab score >0.4737 and were classified as “frail.”

The minimum follow-up time was 1 day, and the maximum was 7.8 years, with an average of 3.4 (SD 2.4) years and a median of 3.2 years. During the follow-up period, 15,138 patients passed away. Of the entire sample of 31,398 patients, 1658 (5.3%) died during hospitalization, 5174 (16.5%) died within the first year after discharge, and 8306 (26.5%) died after the first year and up to the end of the follow-up period.

There were no statistically significant differences between the 45 patients excluded from the sample and the 31,443 patients included in the sample, in terms of age (*P*=.83), gender (*P*=.35), ethnicity (*P*=.80), CCI score (*P*=.46), or length of hospital stay (*P*=.25). In-hospital mortality rates were higher among the 45 patients excluded from the sample (15.6% vs 5.3%, *P*=.02). Differences in mortality rates during the first year after discharge (13.2% vs 17.4%, *P*=.66) and beyond the first year after discharge (39.4% vs 33.8%, *P*=.61) were not statistically significant.

The distribution of patient characteristics according to frailty status groups assessed using FI-Lab scores is presented in Table 2. As the FI-Lab score increased, patients in the group tended to be older (*P*<.001) and have a higher burden of comorbidities, as measured using the CCI (*P*<.001). Male representation was lower in both extreme groups (robust and frail) compared with the other 2 groups (*P*<.001). No differences in FI-Lab scores were found between Jewish and Arab patients.

The length of hospital stay was higher in the robust group than in the mildly prefrail group, but as the severity of frailty increased, a longer hospital stay was observed (*P*<.001).

**Table 2.** Comparison of patient characteristics by frailty status group, assessed using the frailty index based on laboratory tests (FI-Lab) score.

Characteristic		Frailty status group								*P* value
		Robust (n=8628)		Mildly prefrail (n=6335)		Moderately prefrail (n=9330)		Frail (n=7105)		
Gender, n (%)										<.001
Male		3975 (46.1)		3113 (49.1)		4640 (49.7)		3324 (46.8)		
Female		4653 (53.9)		3222 (50.9)		4690 (50.3)		3781 (53.2)		
Age (years), mean (SD)		75.4 (7.6)		77.2 (8.0)		78.4 (8.3)		79.8 (8.5)		<.001
Age (years), median (range)		74 (65-107)		77 (65-108)		78 (65-111)		80 (65-111)		<.001
Ethnicity, n (%)										.65
Jewish		7615 (88.4)		5625 (88.9)		8281 (88.8)		6303 (88.8)		
Arab		1004 (11.6)		701 (11.1)		1041 (11.2)		791 (11.2)		
Missing		9 (0.1)		9 (0.1)		8 (0.1)		11 (0.2)		
Total CCI^a^ score, mean (SD)		2.9 (2.4)		3.4 (2.6)		3.9 (2.8)		4.5 (3.1)		<.001
Total CCI score, median (range)		2 (0‐18)		3 (0‐19)		3 (0‐22)		4 (0‐20)		<.001
Mortality, n (%)										<.001
Alive		6083 (70.5)		3772 (59.5)		4240 (45.4)		2165 (30.5)		
Died while hospitalized		105 (1.2)		128 (2)		515 (5.5)		910 (12.8)		
Died in the first year		710 (8.2)		746 (11.8)		1789 (19.2)		1929 (27.1)		
Died after a year		1730 (20.1)		1689 (26.7)		2786 (29.9)		2101 (29.6)		
Hospitalization duration (days), mean (SD)		2.76 (6.1)		2.45 (4.5)		4.0 (7.4)		5.1 (8.8)		<.001

a CCI: Charlson Comorbidity Index.

### Mortality

In-hospital mortality rates within the first year after discharge and beyond that increased with higher FI-Lab scores. For example, in the robust group, in-hospital mortality was 1.2% (105/8628), compared with 2% (128/6335) in the mildly prefrail group, 5.5% (515/9330) in the moderately frail group, and 12.8% (910/7105) in the frail group. Overall, only 2165 patients (2165/7105, 30.5%) in the frail group were alive at the end of the follow-up period, whereas 6083 (70.5%) of the 8628 patients in the robust group survived until the end of the follow-up period (*P*<.001).

Table 3 presents the results of the Cox proportional hazard models for FI-Lab as a predictor of all-cause mortality. In the unadjusted model (Model 1); after adjustment for age and gender (Model 2); after adjustment for age, gender, and CCI (Model 3); and after adjustment for age, gender, CCI, and primary indication for hospitalization (Model 4), FI-Lab, categorized dichotomously into frailty groups (model type a) or used as a continuous variable (model type b), was consistently associated with an increased risk for mortality.

**Table 3.** Cox proportional hazard models of frailty index based on laboratory tests (FI-Lab) for prediction of all-cause mortality.

Model and variables	In-hospital mortality				Mortality during the first year after discharge				Mortality beyond the first year after discharge			
	Hazard ratio (95% CI)			*P* value	Hazard ratio (95% CI)			*P* value	Hazard ratio (95% CI)			*P* value
1a												
FI-Lab score												
Robust	1			—^a^	1			—	1			—
Mildly prefrail	1.67 (1.29-2.16)			<.001	1.47 (1.33-1.63)			<.001	1.51 (1.41-1.61)			<.001
Moderately prefrail	4.65 (3.77-5.74)			<.001	2.62 (2.4-2.85)			<.001	2.07 (1.95-2.2)			<.001
Frail	11.34 (9.27-13.88)			<.001	4.34 (3.98-4.73)			<.001	2.91 (2.73-3.1)			<.001
1b												
FI-Lab score (continuous)	1.06 (1.06-1.06)			<.001	1.04 (1.04-1.04)			<.001	1.03 (1.03-1.03)			<.001
2a												
F1-Lab score												
Robust	1			—	1			—	1			—
Mildly prefrail	1.53 (1.18-1.98)			<.001	1.34 (1.21-1.48)			<.001	1.36 (1.27-1.45)			<.001
Moderately prefrail	4 (3.24-4.94)			<.001	2.23 (2.04-2.43)			<.001	1.76 (1.65-1.87)			<.001
Frail	9.21 (7.51-11.29)			<.001	3.52 (3.23-3.84)			<.001	2.36 (2.22-2.52)			<.001
2b												
F1-Lab score (continuous)	1.06 (1.05-1.06)			<.001	1.03 (1.03-1.03)			<.001	1.02 (1.02-1.02)			<.001
3a												
F1-Lab score												
Robust	1			—	1			—	1			—
Mildly prefrail	1.48 (1.15-1.92)			<.001	1.27 (1.15-1.41)			<.001	1.29 (1.21-1.38)			<.001
Moderately prefrail	3.76 (3.04-4.64)			<.001	2 (1.83-2.18)			<.001	1.6 (1.5-1.7)			<.001
Frail	8.38 (6.83-10.29)			<.001	2.97 (2.72-3.25)			<.001	2.07 (1.94-2.21)			<.001
3b												
F1-Lab score (continuous)	1.06 (1.05-1.06)			<.001	1.03 (1.03-1.03)			<.001	1.02 (1.02-1.02)			<.001
4a												
F1-Lab score												
Robust	1			—	1			—	1			—
Mildly prefrail	1.44 (1.11-1.86)			.01	1.22 (1.1-1.35)			<.001	1.25 (1.17-1.34)			<.001
Moderately prefrail	3.54 (2.86-4.38)			<.001	1.85 (1.7-2.02)			<.001	1.52 (1.43-1.62)			<.001
Frail	7.63 (6.2-9.39)			<.001	2.65 (2.43-2.9)			<.001	1.91 (1.79-2.04)			<.001
4b												
FI-Lab score (continuous)	1.05 (1.05-1.06)			<.001	1.03 (1.02-1.03)			<.001	1.02 (1.02-1.02)			<.001

a Not applicable.

It can be seen that, in all models, the crude model (Model 1); the model adjusted for age and sex (Model 2); the model adjusted for age, sex, and CCI (Model 3); and the model adjusted for age, sex, CCI, and the primary indication for hospitalization (Model 4), an increase in frailty level, as measured using the FI-Lab, was associated with an increase in all-cause mortality during hospitalization, within the first year postdischarge, and throughout the entire follow-up period.

This association was evident when FI-Lab was treated as a categorical variable (robust, mildly prefrail, moderately prefrail, and frail) as well as when it was treated as a continuous variable (per 0.01-point increase in FI-Lab).

For example, in Model 4a (adjusted for age, gender, CCI, and the primary indication for hospitalization), the odds of a patient in the frail group not surviving hospitalization were more than 7 times higher than for those in the robust group (*P*<.001). Similarly, the odds of a patient in the moderately prefrail group dying during hospitalization were more than 3 times higher than for robust patients (*P*<.001). When analyzed as a continuous variable, in Model 4b, every 0.01 increase in the FI-Lab score was associated with a 5.3% increase in in-hospital mortality (*P*<.001), a 2.6% increase in mortality during the first year after discharge (*P*<.001), and a 1.7% increase in mortality by the end of the follow-up period (*P*<.001).

The full multivariable Cox regression results models, including all core confounders (age, gender, CCI, and main reason for hospitalization), are presented in Multimedia Appendix 1 for reference and comprehensive interpretation.

**Figure 1.**
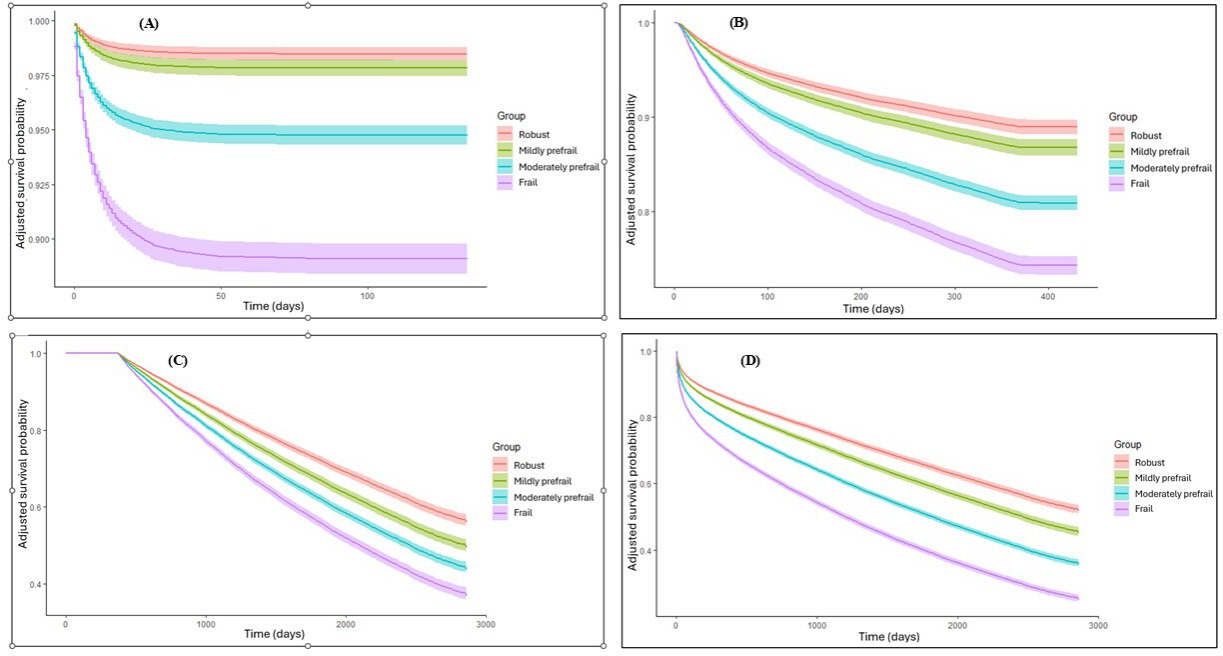
Confounder-adjusted survival curves for all-cause mortality according to frailty status assessed using frailty index based on laboratory tests (FI-Lab) scores (A) during the index hospitalization, (B) during the first year after the index hospitalization, (C) beyond the first year after discharge, and (D) at any time.

Figure 1 presents the adjusted survival curves for age, gender, CCI, and primary indication for hospitalization of the study population during hospitalization (Figure 1A), during the first year after discharge (Figure 1B), beyond the first year (Figure 1C), and any time (Figure 1D), stratified by FI-Lab–based frailty status. In all frail groups, patients had the lowest survival rates in all the examined time frames (all *P*<.001).

**Figure 2.**
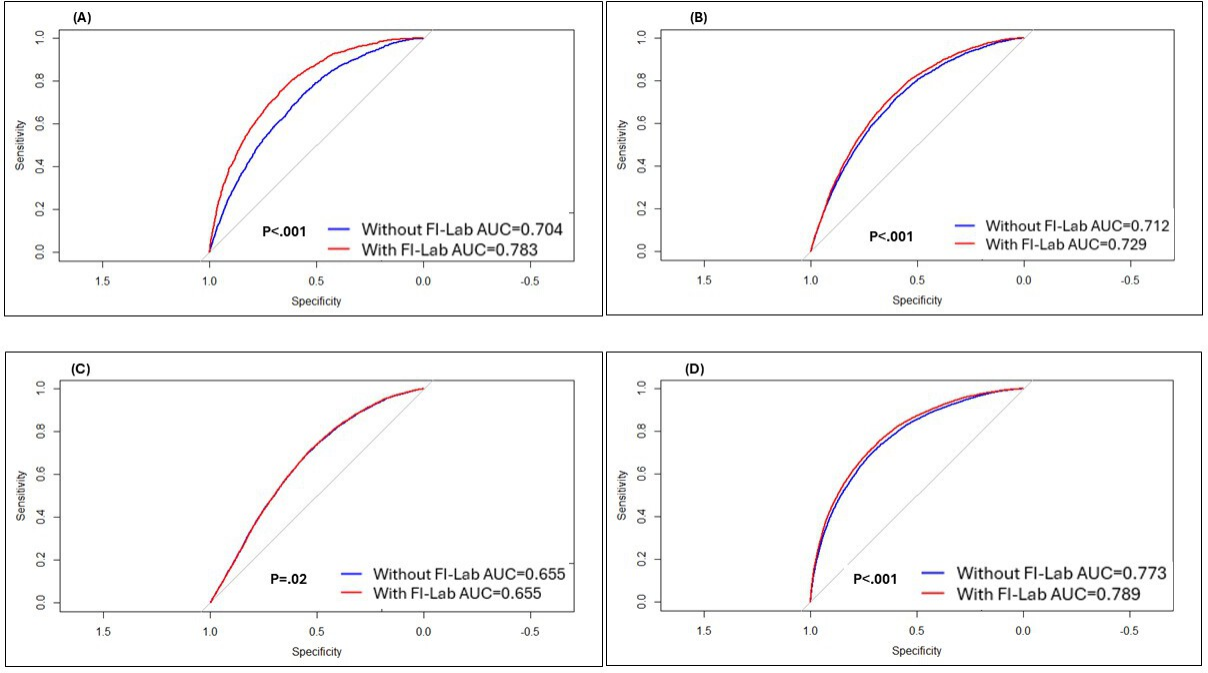
Comparison of receiver operating characteristic (ROC) curves for mortality (A) during the index hospitalization, (B) during the first year after the index hospitalization, (C) beyond the first year after the index hospitalization, and (D) at any time. AUC: area under the curve; FI-Lab: frailty index based on laboratory tests.

According to the ROC analysis (Figure 2), the inclusion of FI-Lab significantly improved the model performance for (Figure 2A) mortality during index hospitalization (AUC=0.783 vs 0.704, *P*<.001). All the other models were statistically significant but with a lower impact of the FI-Lab: (Figure 2B) mortality within the first year after hospitalization (AUC=0.729 vs 0.712, *P*<.001), (Figure 2C) mortality beyond the first year after hospitalization (AUC=0.655 vs 0.655, *P*=.02), and (Figure 2D) mortality at any time (AUC=0.789 vs 0.773, *P*<.001). This reflects the method’s high sensitivity in large datasets and should be interpreted in light of the limited clinical impact of small AUC gains.

## Discussion

In this study, involving a sample of more than 33,000 patients aged 65 years and older who were hospitalized in the internal medicine departments of a tertiary medical center, FI-Lab was found to be an independent prognostic factor for increased mortality, both in the short term and long term, whether analyzed as a dichotomous or continuous variable. These results are consistent with prior research with older hospitalized populations. Multimedia Appendix 2 summarizes 25 studies, all of which demonstrated that FI-Lab is a reliable predictor of mortality in this patient group.

Variations can be seen among the studies in terms of mortality rates, even during comparable periods. For example, in the study by Ding et al [29], each 0.01 increase in FI-Lab was associated with a 3% increase in in-hospital mortality, whereas in the study by Nakashima et al [25], an identical increase in the FI-Lab resulted in a 9.8% increase in in-hospital mortality. This could depend, of course, on the type of study (the first study was retrospective, while the second was prospective); different in-hospital mortality rates (21.6% vs 7.4%), which probably were associated with many variables; the type of patient (patients with sepsis in the first study vs acutely admitted to the geriatric wards in the second study); the number of FI-Lab variables (33 vs 40); and other factors.

Although the association between FI-Lab and all-cause mortality in this study was statistically significant across all time points (during hospitalization, 1-year postdischarge, and throughout the entire follow-up period; see Table 3), the strength of this association declined over time. This is also reflected in the decrease in AUC in the ROC analysis (Figure 2), which was higher during hospitalization than in later time periods.

Additionally, the lower difference in AUC with and without the inclusion of FI-Lab for predicting mortality beyond the first year and for mortality at any time further suggests that the association between FI-Lab and mortality weakens over time. A similar trend has been observed in several other studies in the field (eg, [25,28]). This phenomenon is likely due to the emergence of new variables over time, such as changes in comorbidities, frailty status, and functional status, which may influence mortality and, in turn, alter the relationship between FI-Lab measured at the time of hospitalization and subsequent mortality. A future prospective study that accounts for these evolving variables throughout the follow-up period could help confirm this hypothesis.

The use of FI-Lab in hospitalization offers several notable advantages. As aforementioned, frailty assessment during hospitalization using “classical” models may involve completing a lengthy checklist of data [18]. In some cases, these assessments rely on subjective data (such as a patient’s self-report of physical activity and weight loss) or require the patient’s cooperation (eg, grip strength or walking speed tests) [19]. Therefore, performing frailty assessments using these models can be challenging in a time- and resource-constrained clinical setting.

In contrast, FI-Lab is typically based on laboratory tests that are routinely performed upon admission and, in some cases, supplemented by basic clinical data such as blood pressure and heart rate measurements. As a result, obtaining the data required for FI-Lab calculations generally does not involve additional effort on the part of clinicians [22,24,31]. In cases of acute illness, laboratory results may remain within normal ranges if there is sufficient reserve capacity in the affected organs. A higher FI-Lab score reflects the severity of the damage, number of affected organs and systems, and individual’s level of frailty [40]. FI-Lab likely captures subclinical changes at the organ and system level and may indicate acute or chronic conditions that have the potential to lead to adverse medical outcomes [18]. However, since FI-Lab assesses the current level of acute or chronic accumulated organ dysfunction, it does not necessarily reflect the patient’s comorbidity status prior to the acute illness [25]. In fact, FI-Lab may assess the current state of biological reserves and predict mortality better than comorbidity indices [34]. For these reasons, FI-Lab can serve as a readily available and effective tool for assessing frailty in older hospitalized patients.

However, it is also important to recognize the tool’s limitations. The results of a study by Jäger et al [35] showed that FI-Lab values may vary between the time of hospital admission and subsequent measurements, and these changes significantly impact the prediction of mortality within 6 months and 1 year. Therefore, in cases of prolonged hospitalization, it may be necessary to reassess FI-Lab periodically.

Another significant limitation of the FI-Lab is that it does not assess functional status and cannot replace clinical frailty assessments or comprehensive geriatric evaluations. Relying solely on FI-Lab without a complementary geriatric assessment may lead to significant errors in prognosis prediction and the development of intervention plans [49]. Over-reliance on FI-Lab can, on one hand, prevent patients with high scores from receiving intensive treatment [23] and, on the other, deprive patients with low scores of the support required for frail patients, such as prevention of delirium and functional decline. Therefore, although this method may serve as an initial tool, it *should not be* the sole approach for frailty assessment during hospitalization.

This study has several advantages. First, to our knowledge, it is the largest study conducted among hospitalized patients aged 65 years and older. The applicability of the tool was highlighted by the finding that, among more than 33,000 patients admitted to SUMC during the study period, FI-Lab could not be calculated for only 45 patients. The study included patients treated for a broad spectrum of medical problems across all internal medicine departments of the medical center, minimizing the influence of specific departmental policies on treatment and prognosis. The research is based on data from SUMC’s EMR, which was established nearly 3 decades ago and is considered highly reliable. Additionally, mortality data were obtained through linkage with the Ministry of Interior, ensuring the reliability of these records. Finally, the extended follow-up period in our study represents yet another significant advantage.

This study has several limitations. It was conducted in a single center using a retrospective design, which inherently introduces risks of selection bias and limits the availability of key clinical parameters such as functional, cognitive, and social status, as well as other frailty measures and environmental factors. These data, which are critical for a comprehensive assessment of a patient’s prognosis, are typically not recorded in the medical records of internal medicine departments and could not be retrospectively obtained. Additionally, although the inclusion of a heterogeneous population of hospitalized patients may enhance the generalizability of our findings, it also limits applicability to specific clinical subgroups (eg, patients with pneumonia, sepsis, or myocardial infarction). The primary outcome examined was all-cause mortality, without exploring other clinically relevant outcomes such as delirium, in-hospital falls, institutionalization, readmissions, or length of stay. FI-Lab was assessed only once using laboratory data obtained at admission and during the first days of hospitalization, which may not reflect a consistent time point due to potential changes in lab values over time. Furthermore, the FI-Lab used in this study was based on 19 laboratory variables; some potentially important tests (eg, liver function) were excluded due to inconsistent availability. As demonstrated in prior studies (see Multimedia Appendix 1), there is no consensus on the optimal number or type of variables to include in FI-Lab, with considerable variability across cohorts. Although our primary aim was to evaluate the predictive utility of FI-Lab, we were unable to incorporate other validated frailty measures (eg, such as the 7-point Clinical Frailty Scale [50] or the Hospital Frailty Risk Score [51]) due to data limitations. It is important to note that previous research in the field has reported inconsistent associations between FI-Lab and other frailty indices [18,20,21,25-27,33,34,38,49,52-58].

Finally, although many associations reached statistical significance—likely due to the large sample size—some effect sizes were small and may not be clinically meaningful at the individual level. Nonetheless, such modest associations may have important implications at the population level, reinforcing the potential of FI-Lab as a scalable tool for risk stratification in hospital settings.

Although many of the associations observed in this study reached statistical significance (eg, *P*<.001), this is not unexpected given the large sample size (>31,000 patients). It is important to distinguish between statistical and clinical significance. For example, the HR of 1.017 per unit increase in FI-Lab reflects a modest effect size that may not be clinically meaningful at the individual level. However, at the population level, even small shifts in risk associated with frailty—as captured by laboratory values—may lead to substantial differences in outcomes across large health care systems. These findings support the utility of FI-Lab as a scalable risk stratification tool while also emphasizing the need for cautious interpretation of small effect sizes in clinical decision-making.

In conclusion, this study, conducted with a large cohort of patients aged 65 years and older who were hospitalized for diverse indications in the internal medicine departments of a large tertiary hospital, demonstrated that FI-Lab is an independent predictor of all-cause mortality during and after hospitalization, even after adjusting for multiple covariates, including the primary indication for admission.

FI-Lab may serve as an effective and easily accessible tool, though not a standalone method, for assessing frailty in older hospitalized patients. Automating FI-Lab calculation at the time of hospital admission could provide emergency department clinicians with additional, meaningful information to support clinical decision-making.

## Supplementary material

10.2196/70204 Multimedia Appendix 1 Full Cox proportional hazard models of the frailty index based on laboratory tests (FI-Lab) for predicting all-cause mortality.

10.2196/70204 Multimedia Appendix 2 Summary of 25 studies that examined the association between the frailty index based on laboratory tests (FI-Lab) and mortality.

## Abbreviations

AUC: area under the curve
CCI: Charlson Comorbidity Index
EMR: electronic medical record
FI-Lab: frailty index based on laboratory tests
HR: hazard ratio
ICU: intensive care unit
ROC: receiver operating characteristic
SUMC: Soroka University Medical Center

## References

[1] Clegg A, Young J, Iliffe S, Rikkert MO, Rockwood K (2013). Frailty in elderly people. Lancet.

[2] Bandeen-Roche K, Seplaki CL, Huang J (2015). Frailty in older adults: a nationally representative profile in the United States. J Gerontol A Biol Sci Med Sci.

[3] Bonjour T, Waeber G, Marques-Vidal P (2021). Trends in prevalence and outcomes of frailty in a Swiss university hospital: a retrospective observational study. Age Ageing.

[4] Ligthart-Melis GC, Luiking YC, Kakourou A, Cederholm T, Maier AB, de van der Schueren MAE (2020). Frailty, sarcopenia, and malnutrition frequently (co-)occur in hospitalized older adults: a systematic review and meta-analysis. J Am Med Dir Assoc.

[5] Chong E, Ho E, Baldevarona-Llego J, Chan M, Wu L, Tay L (2017). Frailty and risk of adverse outcomes in hospitalized older adults: a comparison of different frailty measures. J Am Med Dir Assoc.

[6] Hubbard RE, Peel NM, Samanta M, Gray LC, Mitnitski A, Rockwood K (2017). Frailty status at admission to hospital predicts multiple adverse outcomes. Age Ageing.

[7] Romero-Ortuno R, Wallis S, Biram R, Keevil V (2016). Clinical frailty adds to acute illness severity in predicting mortality in hospitalized older adults: an observational study. Eur J Intern Med.

[8] Comans TA, Peel NM, Hubbard RE, Mulligan AD, Gray LC, Scuffham PA (2016). The increase in healthcare costs associated with frailty in older people discharged to a post-acute transition care program. Age Ageing.

[9] García-Nogueras I, Aranda-Reneo I, Peña-Longobardo LM, Oliva-Moreno J, Abizanda P (2017). Use of health resources and healthcare costs associated with frailty: the FRADEA study. J Nutr Health Aging.

[10] Joosten E, Demuynck M, Detroyer E, Milisen K (2014). Prevalence of frailty and its ability to predict in hospital delirium, falls, and 6-month mortality in hospitalized older patients. BMC Geriatr.

[11] Wallis SJ, Wall J, Biram RWS, Romero-Ortuno R (2015). Association of the clinical frailty scale with hospital outcomes. QJM.

[12] Mitnitski AB, Mogilner AJ, Rockwood K (2001). Accumulation of deficits as a proxy measure of aging. ScientificWorldJournal.

[13] Fried LP, Tangen CM, Walston J (2001). Frailty in older adults: evidence for a phenotype. J Gerontol A Biol Sci Med Sci.

[14] Buta BJ, Walston JD, Godino JG (2016). Frailty assessment instruments: systematic characterization of the uses and contexts of highly-cited instruments. Ageing Res Rev.

[15] Basic D, Shanley C (2015). Frailty in an older inpatient population: using the clinical frailty scale to predict patient outcomes. J Aging Health.

[16] Leite HT, Manhães AC, Antunes LA, Chan T, Hajj-Boutros G, Morais JA (2022). The implementation of a geriatrics co-management model of care reduces hospital length of stay. Healthcare (Basel).

[17] Rezaei-Shahsavarloo Z, Atashzadeh-Shoorideh F, Gobbens RJJ, Ebadi A, Ghaedamini Harouni G (2020). The impact of interventions on management of frailty in hospitalized frail older adults: a systematic review and meta-analysis. BMC Geriatr.

[18] Ritt M, Jäger J, Ritt JI, Sieber CC, Gaßmann KG (2017). Operationalizing a frailty index using routine blood and urine tests. Clin Interv Aging.

[19] Jin X, Ren Y, Shao L (2021). Prevalence of frailty and prediction of mortality in Chinese cancer patients using a frailty index-based clinical algorithm-a multicentre study. Cancer Med.

[20] Howlett SE, Rockwood MRH, Mitnitski A, Rockwood K (2014). Standard laboratory tests to identify older adults at increased risk of death. BMC Med.

[21] Ellis HL, Wan B, Yeung M (2020). Complementing chronic frailty assessment at hospital admission with an electronic frailty index (FI-Laboratory) comprising routine blood test results. CMAJ.

[22] Kim Y, Song K, Kang CM, Lee H (2022). Impact of preoperative laboratory frailty index on mortality and clinical outcomes in older surgical patients with cancer. Sci Rep.

[23] Lim A, Choi M, Jang Y, Lee H (2022). Preoperative frailty based on laboratory data and postoperative health outcomes in patients undergoing coronary artery bypass graft surgery. Heart Lung.

[24] Nagae M, Umegaki H, Nakashima H, Nishiuchi T (2025). FI-Lab in the emergency department and adverse outcomes among acutely hospitalized older adults. Arch Gerontol Geriatr.

[25] Nakashima H, Nagae M, Komiya H (2023). Combined use of the Clinical Frailty Scale and laboratory tests in acutely hospitalized older patients. Aging Clin Exp Res.

[26] Li M, She Q, Tu J (2023). Association of frailty with clinical outcomes in chronic obstructive pulmonary disease: a retrospective longitudinal cohort study. Heliyon.

[27] Ysea-Hill O, Gomez CJ, Mansour N (2022). The association of a frailty index from laboratory tests and vital signs with clinical outcomes in hospitalized older adults. J Am Geriatr Soc.

[28] Bai W, Hao B, Xu L, Qin J, Xu W, Qin L (2022). Frailty index based on laboratory tests improves prediction of short-and long-term mortality in patients with critical acute myocardial infarction. Front Med (Lausanne).

[29] Ding H, Li X, Zhang X, Li J, Li Q (2024). The association of a frailty index derived from laboratory tests and vital signs with clinical outcomes in critical care patients with septic shock: a retrospective study based on the MIMIC-IV database. BMC Infect Dis.

[30] Gu JJ, Liu Q, Zheng LJ (2021). A frailty assessment tool to predict in-hospital mortality in patients with acute exacerbations of chronic obstructive pulmonary disease. Int J Chron Obstruct Pulmon Dis.

[31] Veronese N, Briganò V, Ciriminna S (2024). Prognostic value of a laboratory index of frailty in older patients hospitalized for COVID-19: the COMEPA study. J Frailty Aging.

[32] Zhao H, Tu J, She Q (2023). Prognostic significance of frailty in hospitalized elderly patients with community-acquired pneumonia: a retrospective cohort study. BMC Geriatr.

[33] Guan L, Soh CH, Reijnierse EM, Lim WK, Maier AB (2022). Association of a modified laboratory frailty index with adverse outcomes in geriatric rehabilitation inpatients: RESORT. Mech Ageing Dev.

[34] Hao B, Xu W, Gao W (2023). Association between frailty assessed using two electronic medical record-based frailty assessment tools and long-term adverse prognosis in older critically ill survivors. J Nutr Health Aging.

[35] Jäger J, Sieber CC, Gaßmann KG, Ritt M (2019). Changes of a frailty index based on common blood and urine tests during a hospital stay on geriatric wards predict 6-month and 1-year mortality in older people. Clin Interv Aging.

[36] Sohn B, Choi JW, Hwang HY, Jang MJ, Kim KH, Kim KB (2019). Frailty index is associated with adverse outcomes after aortic valve replacement in elderly patients. J Korean Med Sci.

[37] Wang S, Wang L, Wang Y (2024). Association between frailty index based on laboratory tests and all-cause mortality in critically ill patients with heart failure. ESC Heart Fail.

[38] Engvig A, Wyller TB, Skovlund E (2022). Association between clinical frailty, illness severity and post-discharge survival: a prospective cohort study of older medical inpatients in Norway. Eur Geriatr Med.

[39] Kim CH, Kang Y, Kim JS, Sohn SH, Hwang HY (2022). Association between the frailty index and clinical outcomes after coronary artery bypass grafting. J Chest Surg.

[40] Klausen HH, Petersen J, Bandholm T (2017). Association between routine laboratory tests and long-term mortality among acutely admitted older medical patients: a cohort study. BMC Geriatr.

[41] Wang Y, Zhang R, Shen Y, Su L, Dong B, Hao Q (2019). Prediction of chemotherapy adverse reactions and mortality in older patients with primary lung cancer through frailty index based on routine laboratory data. Clin Interv Aging.

[42] Charlson ME, Pompei P, Ales KL, MacKenzie CR (1987). A new method of classifying prognostic comorbidity in longitudinal studies: development and validation. J Chronic Dis.

[43] Schoenfeld DA (1983). Sample-size formula for the proportional-hazards regression model. Biometrics.

[44] R Core Team (2024). R: a language and environment for statistical computing version 443. R Foundation for Statistical Computing.

[45] Denz R AdjustedCurves: confounder-adjusted survival curves and cumulative incidence functions. The Comprehensive R Archive Network.

[46] Therneau TM, Lumley T, Atkinson E, Crowson C Survival: survival analysis. The Comprehensive R Archive Network.

[47] Wickham H (2016). Ggplot2: Elegant Graphics for Data Analysis.

[48] Kassambara A, Kosinski M, Biecek P, Fabian S Survminer: drawing survival curves using “ggplot2”. The Comprehensive R Archive Network.

[49] Nakashima H, Watanabe K, Komiya H (2024). Frailty index based on common laboratory tests for patients starting home-based medical care. J Am Med Dir Assoc.

[50] Rockwood K, Song X, MacKnight C (2005). A global clinical measure of fitness and frailty in elderly people. CMAJ.

[51] Gilbert T, Neuburger J, Kraindler J (2018). Development and validation of a Hospital Frailty Risk Score focusing on older people in acute care settings using electronic hospital records: an observational study. Lancet.

[52] Blodgett JM, Pérez-Zepeda MU, Godin J (2022). Frailty indices based on self-report, blood-based biomarkers and examination-based data in the Canadian Longitudinal Study on Aging. Age Ageing.

[53] Blodgett JM, Theou O, Howlett SE, Rockwood K (2017). A frailty index from common clinical and laboratory tests predicts increased risk of death across the life course. Geroscience.

[54] Blodgett JM, Theou O, Howlett SE, Wu FCW, Rockwood K (2016). A frailty index based on laboratory deficits in community-dwelling men predicted their risk of adverse health outcomes. Age Ageing.

[55] Chao CT, Huang JW, Chiang CK, Hung KY, COhort of GEriatric Nephrology in NTUH (COGENT) Study Group (2020). Applicability of laboratory deficit-based frailty index in predominantly older patients with end-stage renal disease under chronic dialysis: a pilot test of its correlation with survival and self-reported instruments. Nephrology (Carlton).

[56] Mitnitski A, Collerton J, Martin-Ruiz C (2015). Age-related frailty and its association with biological markers of ageing. BMC Med.

[57] Yang M, Zhuo Y, Hu X, Xie L (2018). Predictive validity of two frailty tools for mortality in Chinese nursing home residents: frailty index based on common laboratory tests (FI-Lab) versus FRAIL-NH. Aging Clin Exp Res.

[58] Rockwood K, McMillan M, Mitnitski A, Howlett SE (2015). A frailty index based on common laboratory tests in comparison with a clinical frailty index for older adults in long-term care facilities. J Am Med Dir Assoc.

