# Supplementary material for: Association Between the Frailty Index Based on Laboratory Tests and All-Cause Mortality in Hospitalized Older Adults: Retrospective Cohort Study
Source: JMIR Aging. 2025 Sep 10;8:e70204. doi: 10.2196/70204 (PMC12422745; doi:10.2196/70204)
Supplement: Multimedia Appendix 1 [file aging-v8-e70204-s001.docx]

**Full Cox proportional hazard models of FI-LAB for predicting of all-cause mortality**

|  | | **In-hospital mortality** | | | | **Mortality during the first year after discharge** | | | | **Mortality beyond the first year after discharge** | | | |  |  |
| --- | --- | --- | --- | --- | --- | --- | --- | --- | --- | --- | --- | --- | --- | --- | --- |
| **Model** | **Variables** | **Hazard Ratio** | **95.0% CI** | | **Pvalue** | **Hazard Ratio** | **95.0% CI** | | **Pvalue** | **Hazard Ratio** | **95.0% CI** | | **Pvalue** |  |  |
|  |  |  | **Lower** | **Upper** |  |  | **Lower** | **Upper** |  |  | **Lower** | **Upper** |  |  |  |
| 1a | **FI-LAB score:** |  |  |  |  |  |  |  |  |  |  |  |  |  |  |
|  | Robust | 1.000 |  |  |  | 1.000 |  |  |  | 1.000 |  |  |  |  |  |
|  | Mildly pre-frail | 1.672 | 1.292 | 2.164 | 0.000 | 1.473 | 1.329 | 1.632 | 0.000 | 1.508 | 1.410 | 1.613 | <.001 |  |  |
|  | Moderately pre-frail | 4.653 | 3.772 | 5.739 | 0.000 | 2.615 | 2.397 | 2.853 | 0.000 | 2.074 | 1.953 | 2.202 | <.001 |  |  |
|  | Frail | 11.341 | 9.266 | 13.880 | 0.000 | 4.338 | 3.980 | 4.728 | 0.000 | 2.913 | 2.733 | 3.104 | <.001 |  |  |
| 1b | **FI-Lab (continuous)** | 1.060 | 1.057 | 1.063 | <.001 | 1.037 | 1.035 | 1.039 | <.001 | 1.027 | 1.025 | 1.028 | <.001 |  |  |
| 2a | **FI-LAB score:** |  |  |  |  |  |  |  |  |  |  |  |  |  |  |
|  | Robust | 1.000 |  |  |  | 1.000 |  |  |  | 1.000 |  |  |  |  |  |
|  | Mildly pre-frail | 1.527 | 1.179 | 1.977 | 0.001 | 1.338 | 1.207 | 1.483 | 0.000 | 1.356 | 1.267 | 1.450 | <.001 |  |  |
|  | Moderately pre-frail | 3.999 | 3.239 | 4.937 | 0.000 | 2.229 | 2.042 | 2.433 | 0.000 | 1.755 | 1.652 | 1.865 | <.001 |  |  |
|  | Frail | 9.206 | 7.509 | 11.286 | 0.000 | 3.519 | 3.225 | 3.839 | 0.000 | 2.363 | 2.215 | 2.520 | <.001 |  |  |
|  | **Gender (Male)** | 1.034 | 0.938 | 1.140 | 0.497 | 1.130 | 1.070 | 1.194 | 0.000 | 1.230 | 1.178 | 1.284 | <.001 |  |  |
|  | **Age (Years)** | 1.049 | 1.043 | 1.055 | 0.000 | 1.055 | 1.052 | 1.059 | 0.000 | 1.071 | 1.068 | 1.074 | <.001 |  |  |
| 2b | **FI-Lab (continuous)** | 1.057 | 1.053 | 1.060 | <.001 | 1.033 | 1.031 | 1.034 | <.001 | 1.022 | 1.020 | 1.023 | <.001 |  |  |
|  | **Gender (Male)** | 1.039 | 0.943 | 1.145 | 0.443 | 1.130 | 1.070 | 1.194 | <.001 | 1.231 | 1.179 | 1.285 | <.001 |  |  |
|  | **Age (Years)** | 1.049 | 1.043 | 1.055 | <.001 | 1.055 | 1.051 | 1.058 | <.001 | 1.071 | 1.068 | 1.073 | <.001 |  |  |
|  |  |  |  |  |  |  |  |  |  |  |  |  |  |  |  |
|  |  |  |  |  |  |  |  |  |  |  |  |  |  |  |  |
|  |  |  |  |  |  |  |  |  |  |  |  |  |  |  |  |
| 3a | **FI-LAB score:** |  |  |  |  |  |  |  |  |  |  |  |  |  |  |
|  | Robust | 1.000 |  |  |  | 1.000 |  |  |  | 1.000 |  |  |  |  |  |
|  | Mildly pre-frail | 1.483 | 1.145 | 1.921 | 0.003 | 1.269 | 1.145 | 1.407 | 0.000 | 1.292 | 1.208 | 1.382 | <.001 |  |  |
|  | Moderately pre-frail | 3.756 | 3.040 | 4.640 | 0.000 | 1.995 | 1.827 | 2.178 | 0.000 | 1.598 | 1.504 | 1.698 | <.001 |  |  |
|  | Frail | 8.379 | 6.825 | 10.288 | 0.000 | 2.973 | 2.722 | 3.247 | 0.000 | 2.071 | 1.941 | 2.211 | <.001 |  |  |
|  | **Gender (Male)** | 0.980 | 0.889 | 1.081 | 0.691 | 1.021 | 0.966 | 1.079 | 0.463 | 1.129 | 1.081 | 1.180 | <.001 |  |  |
|  | **Age (Years)** | 1.050 | 1.044 | 1.056 | 0.000 | 1.055 | 1.051 | 1.058 | 0.000 | 1.070 | 1.067 | 1.073 | <.001 |  |  |
|  | **CCI score** | 1.062 | 1.045 | 1.079 | 0.000 | 1.119 | 1.109 | 1.128 | 0.000 | 1.112 | 1.104 | 1.120 | <.001 |  |  |
| 3b | **FI-Lab (continuous)** | 1.055 | 1.051 | 1.058 | <.001 | 1.029 | 1.027 | 1.030 | <.001 | 1.019 | 1.017 | 1.020 | <.001 |  |  |
|  | **Gender (Male)** | 0.986 | 0.894 | 1.088 | 0.785 | 1.021 | 0.966 | 1.079 | 0.463 | 1.130 | 1.082 | 1.181 | <.001 |  |  |
|  | **Age (Years)** | 1.049 | 1.043 | 1.055 | <.001 | 1.054 | 1.051 | 1.058 | <.001 | 1.069 | 1.066 | 1.072 | <.001 |  |  |
|  | **CCI score** | 1.057 | 1.041 | 1.074 | <.001 | 1.117 | 1.108 | 1.127 | <.001 | 1.112 | 1.104 | 1.12 | <.001 |  |  |
| 4a | **FI-LAB score:** |  |  |  |  |  |  |  |  |  |  |  |  |  |  |
|  | Robust | 1.000 |  |  |  | 1.000 |  |  |  | 1.000 |  |  |  |  |  |
|  | Mildly pre-frail | 1.435 | 1.108 | 1.859 | 0.006 | 1.220 | 1.101 | 1.353 | 0.000 | 1.254 | 1.173 | 1.342 | 0.000 |  |  |
|  | Moderately pre-frail | 3.541 | 2.863 | 4.378 | 0.000 | 1.853 | 1.696 | 2.024 | 0.000 | 1.521 | 1.430 | 1.617 | 0.000 |  |  |
|  | Frail | 7.628 | 6.201 | 9.385 | 0.000 | 2.652 | 2.426 | 2.900 | 0.000 | 1.908 | 1.786 | 2.039 | 0.000 |  |  |
|  | **Gender (Male)** | 0.999 | 0.906 | 1.102 | 0.986 | 1.046 | 0.990 | 1.106 | 0.111 | 1.149 | 1.099 | 1.200 | 0.000 |  |  |
|  | **Age (Years)** | 1.049 | 1.043 | 1.055 | 0.000 | 1.054 | 1.050 | 1.057 | 0.000 | 1.069 | 1.066 | 1.072 | 0.000 |  |  |
|  | **CCI score** | 1.061 | 1.044 | 1.077 | 0.000 | 1.118 | 1.109 | 1.128 | 0.000 | 1.112 | 1.104 | 1.120 | 0.000 |  |  |
|  | **Hospitalization primary reason:** |  |  |  |  |  |  |  |  |  |  |  |  |  |  |
|  | Cardiovascular | 0.574 | 0.495 | 0.666 | 0.000 | 0.668 | 0.618 | 0.721 | 0.000 | 0.868 | 0.819 | 0.919 | 0.000 |  |  |
|  | Musculoskeletal | 0.601 | 0.501 | 0.721 | 0.000 | 0.517 | 0.468 | 0.572 | 0.000 | 0.674 | 0.629 | 0.722 | 0.000 |  |  |
|  | Respiratory | 0.981 | 0.840 | 1.146 | 0.809 | 0.951 | 0.869 | 1.039 | 0.266 | 1.190 | 1.109 | 1.277 | 0.000 |  |  |
|  | Fever | 0.753 | 0.643 | 0.882 | 0.000 | 0.869 | 0.795 | 0.950 | 0.002 | 1.066 | 0.993 | 1.144 | 0.077 |  |  |
| 4b | **FI-Lab (continuous)** | 1.053 | 1.049 | 1.056 | 0.000 | 1.026 | 1.024 | 1.028 | 0.000 | 1.017 | 1.015 | 1.018 | 0.000 |  |  |
|  | **Gender (Male)** | 1.004 | 0.910 | 1.108 | 0.933 | 1.046 | 0.989 | 1.105 | 0.115 | 1.149 | 1.100 | 1.201 | 0.000 |  |  |
|  | **Age (Years)** | 1.049 | 1.043 | 1.055 | 0.000 | 1.053 | 1.050 | 1.057 | 0.000 | 1.069 | 1.066 | 1.071 | 0.000 |  |  |
|  | **CCI score** | 1.055 | 1.039 | 1.072 | 0.000 | 1.116 | 1.107 | 1.126 | 0.000 | 1.111 | 1.103 | 1.119 | 0.000 |  |  |
|  | **Hospitalization primary reason:** |  |  |  |  |  |  |  |  |  |  |  |  |  |  |
|  | Cardiovascular | 0.593 | 0.511 | 0.688 | 0.000 | 0.672 | 0.622 | 0.726 | 0.000 | 0.870 | 0.821 | 0.922 | 0.000 |  |  |
|  | Musculoskeletal | 0.619 | 0.516 | 0.743 | 0.000 | 0.524 | 0.474 | 0.579 | 0.000 | 0.678 | 0.633 | 0.727 | 0.000 |  |  |
|  | Respiratory | 1.016 | 0.870 | 1.188 | 0.839 | 0.959 | 0.877 | 1.049 | 0.362 | 1.197 | 1.116 | 1.284 | 0.000 |  |  |
|  | Fever | 0.737 | 0.630 | 0.863 | 0.000 | 0.860 | 0.787 | 0.940 | 0.001 | 1.056 | 0.983 | 1.134 | 0.134 |  |  |
|  |  |  |  |  |  |  |  |  |  |  |  |  |  |  |  |
| * continuous variables (0.01 units) | | |  |  |  |  |  |  |  |  |  |  |  |  |  |
|  |  |  |  |  |  |  |  |  |  |  |  |  |  |  |  |
|  |  |  |  |  |  |  |  |  |  |  |  |  |  |  |  |
|  | |  |  |  |  |  |  |  |  |  |  |  |  |  |  |
