# Supplementary material for: Association Between the Frailty Index Based on Laboratory Tests and All-Cause Mortality in Hospitalized Older Adults: Retrospective Cohort Study
Source: JMIR Aging. 2025 Sep 10;8:e70204. doi: 10.2196/70204 (PMC12422745; doi:10.2196/70204)
Supplement: Multimedia Appendix 2 [file aging-v8-e70204-s002.docx]

**Summary of 25 studies that examined the association between FI-LAB and mortality**

| **N of study** | **1** | **2** | **3** | **4** | **5** | **6** | **7** | **8** | **9** |
| --- | --- | --- | --- | --- | --- | --- | --- | --- | --- |
| **Authors** | Bai W et al.[28] | Ding H et al.[29] | Ellis HL et al.[21] | Engvig A et al.[38] | Gu JJ at al.[30] | Guan L et al.[33] | Hao B et al.[34] | Huang S et al.[59] | Jager J et al.[35] |
| **Publication year** | 2022 | 2024 | 2020 | 2021 | 2021 | 2022 | 2023 | 2002 | 2019 |
| **Country** | China | China | UK | Norway | China | Australia | USA | China | Germany |
| **Study design** | Retrospective | Retrospective | Prospective | Prospective | Retrospective | Prospective | Retrospective | Retrospective | Prospective |
| **Setting** | Hospital | Hospital | Hospital | Hospital | Hospital | Hospital | Hospital | Hospital | Hospital |
| **Participants** | Patients with critical acute MI | Septic patients admitted to ICU | Admitted to hospital (acute geriatric medical service) | Admitted to hospital (acute geriatric and general medical) | Admitted to hospital due to exacerbation of COPD | Geriatric rehabilitation inpatients | ICU admission | Patients with Community-Aqured Pneumonia | Hospitalized in the geriatric wards |
| **N of participants** | 2159 | 9219 | 2254 | 195 | 154 | 1819 | 9082 | 627 | 500 |
| **Age (years)** | >18, median 72 | ≥18; survivor =median 67.7, non-survivor= median 70.3 | ≥65; 84.6 ±14.0 | ≥ 75; 86±5.7 | ≥60; 79.7±8.4 | ≥70; median 83.3 | ≥65; 77.2±7.8 | ≥60; median 80 | ≥65; 82.8±6.2 |
| **Females (%)** | 39.9 | 41.1 | 55.3 | 63 | 29.9 | 56.6 | 47.7 | 39.2 | 67.4 |
| **Follow-up time** | 1 year | In hospital | 21 months for all outcomes, 44 months for mortality | 20 months | In-hospital | 3 months | 1 year | NA | 1 year |
| **FI-LAB N of deficits** | 33 | 33 | 27 | 14 | 23 | 77 | 33 | 44 | 21 |
| **N of clinical deficits in FI-LAB** | 3 | 3 | 0 | 0 | 2 | 0 | 3 | 0 | 0 |
| **Minimal presented FI-LAB deficits for inclusion (%)** | 80 | 64 | 70 | NA | 70 | NA | 80 | NA | 80 |
| **FI-LAB score** | median 0.45 | Surivor median=0.43,non-survivor median=0.54. | median 0.3 | 0.37±0.15; median=0.36 | survivors= 0.29±0.10; in non-survivors=0.51±0.13 | median= 0.31 | 0.4±0.12 | NA | 0.33±0.15 median=0.33 |
| **In-hospital mortality (%)** | 22.1% | 21.6% | 8.1% |  |  |  |  |  |  |
| **Increase in in-hospital mortality (for each 0.01 or 0.1 of FI-LAB score)** | 0.01=6% | 0.01=3% |  |  |  |  |  |  |  |
| **OR in-hospital mortality** |  | Q4 vs Q1:OR=2.91 |  |  | OR=8.705 |  |  |  |  |
| **30 days mortality** |  |  |  |  |  |  |  |  |  |
| **↑ 30-days mortality for↑ each 0.01or 0.1 FILAB score** |  |  |  |  |  |  |  |  |  |
| **OR 30-days mortality** |  |  |  |  |  |  |  |  |  |
| **1 year mortality (%)** | 41.6% |  |  | 25.0% |  |  | 28.5% |  | 23.1% |
| **Increase in 1-year mortality (for each 0.01 or 0.1 of FI-LAB score)** | 0.01=5% |  |  |  |  | 0.1=32% | 0.01=3% |  | 6 months:0.01=4.9%; 1 year:0.01=4.8% |
| **OR 1 year mortality** |  |  |  |  |  |  | Frail (FI-LAB>0.48) vs. robust (FI-LAB<0.36) OR=2.3 |  |  |
| **Mortality during other follow-up period** |  |  | 44 months=56.4% | 20 months=32.0% |  |  |  | 46.9% |  |
| **Increase in mortality in other follow-up period for each 0.01 or 0.1 of FI-LAB score** |  |  | 44 months:0.1=45% | 20 months:0.01=3% |  |  |  |  |  |
| **OR for mortality in other follow-up period** |  |  |  |  |  |  |  | Frail (FILAB≥0.35 vs. robust (FILAB<0.2) OR=2.88 |  |
| **Performance of FI-Lab in mortality prediction (AUC)** |  |  |  |  | In-hospital=0.906 |  |  |  | 6 months :0.720; 1 year-0.720 |

| **N of study** | **10** | **11** | **12** | **13** | **14** | **15** | **16** | **17** | **18** |
| --- | --- | --- | --- | --- | --- | --- | --- | --- | --- |
| **Authors** | Jin X et al.[19] | Kim CH et al.[39] | Kim Y et al.[22] | Klausen HH et al.[40] | Li M et al.[26] | Nagae M et al.[24] | Nakashima H et al.[25] | Ritt M et al.[18] | Soh CH et al.[60] |
| **Publication year** | 2021 | 2022 | 2022 | 2017 | 2023 | 2024 | 2023 | 2017 | 2022 |
| **Country** | China | South Korea | South Korea | Denmark | China | Japan | Japan | Germany | Australia |
| **Study design** | Prospective | Prospective | Retrospective | Prospective | Retrospective | Retrospective | Prospective | Prospective | Prospective |
| **Setting** | Hospital | Hospital | Hospital | Hospital | Hospital | ER | Hospital | Hospital | Hospital |
| **Participants** | Cancer patients | Patient after CABG | Patient after cancer surgery | Acutely admitted medical patients | Patients with COPD | Admitted to hospital | Acutely admitted medical patients of geriatric wards | Hospitalized in the geriatric wards | Geriatric rehabilitation inpatients |
| **N of participants** | 2959 | 508 | 9015 | 4005 | 826 | 872 | 378 | 306 | 1819 |
| **Age (years)** | >20; 55.8±11.7 | 67.3±9.7 | ≥65; 72.3±5.3 | ≥65; median 79.4 | median 74.0 | ≥65; 80.9±7.7 | ≥65; 85.2±5.8 | ≥65; 82.9±6.4 | ≥70, median 83.3 |
| **Females (%)** | 43.5 | 22 | 34.5 | 57.6 | 16.2 | 47.4 | 59.3 | 67.6 | 56.6 |
| **Follow-up time** | 5 years | 3 years | 5 years | 3 years | 30 days | In hospital | 3 months | 1 year | 1 year |
| **FI-LAB N of deficits** | 22 | 32 | 32 | 17 | NA | 24 | 23 | 23 | 77 |
| **N of clinical deficits in FI-LAB** | 0 | 3 | 3 | 0 | NA | 0 | 0 | 0 | 0 |
| **Minimal presented FI-LAB deficits for inclusion (%)** | NA | NA | 70 | 59 | NA | 50 | 50 | 80 | NA |
| **FI-LAB score** | median-0.227. | 0.21±0.11 | 0.2±0.1 |  | NA | 0.52±0.14 | median 0.43 | median 0.34 | 0.31 |
| **In-hospital mortality (%)** |  |  |  |  |  | 9.7% | 7.4% |  |  |
| **Increase in in-hospital mortality (for each 0.01 or 0.1 of FI-LAB score)** |  |  |  |  |  | 0.1=91% | 0.1=98% |  |  |
| **OR in-hospital mortality** |  |  |  |  |  |  |  |  |  |
| **30 days mortality** |  | 6% |  |  | 8.50% |  |  |  |  |
| **↑30-days mortality for each ↑ 0.01or 0.1 in FILAB score** |  |  |  |  |  |  |  |  |  |
| **OR 30-days mortality** |  |  |  |  | 4.3% in robust (FiLAB≤0.2) vs. 11.2% in frail (FiLAB≥0⋅35) |  |  |  |  |
| **1 year mortality (%)** |  | 4.0% |  |  |  |  |  | 20.4% | 17.1% |
| **Increase in 1-year mortality (for each ↑ of 0.01 or 0.1 in FI-LAB score)** |  |  |  |  |  |  |  | 0.01=7.1% | 0.1=18% |
| **OR 1 year mortality** |  |  |  |  |  |  |  |  |  |
| **Mortality during other follow-up period** | 10 years: Robust (FI-LAB<0.21)=14.3%; Frail (FI-LAB≥0.21)=26% | 3 years= 11.3% | 5 years=8% |  |  |  | 3 months =15.8% | 6 month mort=15.4% |  |
| **Increase in mortality in other follow-up period for each increase of 0.01 or 0.1 of FI-LAB score** |  | 3 years: 0.01=4.2% |  |  |  |  | 3 months: 0.1=62% | 6 months: 0.01 =7.2% |  |
| **OR for mortality in other follow-up period** |  |  | Frail (FI-LAB>0.4) vs. robust (FI-LAB<0.25):HR=4.29 | Q4 vs Q1 OR=3.48 |  |  |  |  |  |
| **Performance of FI-Lab in mortality prediction (AUC)** |  |  |  |  | 30 days=0.832 | In-hospital=0.720 |  | 6 months=0.765; 1 year= 0.769 | 1 year =0.581 |

| **N of study** | **19** | **20** | **21** | **22** | **23** | **24** | **25** |
| --- | --- | --- | --- | --- | --- | --- | --- |
| **Authors** | Sohn B et al.[36] | Veronese N et al.[31] | Wang S et al.[37] | Wang Y et al.[41] | Ysea-Hill O et al.[27] | Zan YM et al.[61] | Zhao H et al.[32] |
| **Publication year** | 2019 | 2024 | 2024 | 2019 | 2022 | 2023 | 2023 |
| **Country** | South Korea | Italy | USA | China | USA | China | China |
| **Study design** | Retrospective | Prospective | Retrospective | Retrospective | Retrospective | Retrospective | Retrospective |
| **Setting** | Hospital | Hospital | Hospital | Hospital | Hospital | Hospital | Hospital |
| **Participants** | Patients underwent surgical aortic valve replacement | Hospitalized with COVID19 | Clinically severe CHF | Patients with primary lung cancer | Hospitalized Veterans | Older patients with Community-Aqured Pneumonia | Patients with Community-Aqured Pneumonia |
| **N of participants** | 154 | 376 | 3021 | 1020 | 1407 | 495 | 1164 |
| **Age (years)** | ≥75, 78.7±3.6 | >18; 65±17 | >18; median=74.3 | ≥60; median=65 | ≥60; 72.7±9.0 | ≥65; 78.7±8.3 | ≥65; median 77 |
| **Females (%)** | 49.3 | 46.3 | 43 | 28.6 | 3.9 | 36.4 | 37.6 |
| **Follow-up time** | 15 years | In hospital | 1 year | 3.9 years | 1 year | 30 days | 33.9 months |
| **FI-LAB N of deficits** | 32 | 40 | 33 | 44 | 31 | 29 | 44 |
| **N of clinical deficits in FI-LAB** | 4 | 0 | 3 | 0 | 4 | 0 | 0 |
| **Minimal presented FI-LAB deficits for inclusion (%)** | NA | 75 | 80 | NA | NA | NA | 70 |
| **FI-LAB score** | NA | median 0.54 | median=0.48 | median=0.14 | 0.26±0.12 | median 0.35 |  |
| **In-hospital mortality (%)** |  |  |  |  | 1.8% |  |  |
| **Increase in in-hospital mortality (for each ↑ of 0.01 or 0.1 in FI-LAB score)** |  | 0.01=8.4 |  |  |  |  |  |
| **OR in-hospital mortality** |  | For FI-LAB>0.54 vs. FI-LAV≤0.54 OR=5.1 |  |  | Frail (FI-LAB>0.40) vs. Robust (FI-LAB<0.25) OR=23.6 |  | Frail (FI-LAB≥0.35) vs. Robust (FI-LAB<0.2) OR=5.01 |
| **30 days mortality** | 3.9% |  | 27.7% |  |  |  |  |
| **↑30-days mortality for each ↑ of 0.01or 0.1 in FILAB score** |  |  | 0.01=2% |  |  | 0.01=6% |  |
| **OR 30-days mortality** |  |  | Q4 vs. Q1 OR=1.66 |  |  |  |  |
| **1 year mortality (%)** |  |  | 46.3% |  | 14.8% |  |  |
| **Increase in 1-year mortality (for each ↑ of 0.01 or 0.1 in FI-LAB score)** |  |  | 0.01=2% |  |  |  |  |
| **OR for 1 year mortality** |  |  | Q4 vs. Q1 OR=1.48 |  | Frail (FI-LAB>0.40) vs. Robust (FI-LAB<0.25) OR=4.8 |  |  |
| **Mortality during other follow-up period** | 5 years=16.7%, 10 years=41%; 15 years=58.4% |  |  | 3.9 years=60.1% |  |  | 35.1% |
| **Increase in mortality in other follow-up period for each 0.01 or 0.1 of FI-LAB score** |  |  |  | 3.9 years=0.01=2% |  |  | 33.9 months: 0.1=58% |
| **OR for mortality in other follow-up period** | 15 years OR=1.075 |  |  |  |  |  | 33.9 month: Frail (FI-LAB≥0.35) vs. Robust (FI-LAB<0.2) OR=3.61 |
| **Performance of FI-Lab in mortality prediction (AUC)** |  | In-hospital=0.910 |  |  | In-hospital=0.840; 30 days mortality=0.780; 6-months mortality=0.710; 1-year mortality=0.680 | 1 month=0.783 | 33.9 months=0.750 |
